# *Legionella* Persistence in Manufactured Water Systems: Pasteurization Potentially Selecting for Thermal Tolerance

**DOI:** 10.3389/fmicb.2017.01330

**Published:** 2017-07-19

**Authors:** Harriet Whiley, Richard Bentham, Melissa H. Brown

**Affiliations:** College of Science and Engineering, Flinders University, Bedford Park SA, Australia

**Keywords:** *Legionella*, Legionnaires disease, *L. pneumophila*, warm water, heat shock, thermal disinfection, drinking water, opportunistic pathogens

## Abstract

*Legionella* is an opportunistic waterborne pathogen of increasing public health significance. Pasteurization, otherwise known as super-heat and flush (increasing water temperature to above 70°C and flushing all outlets), has been identified as an important mechanism for the disinfection of *Legionella* in manufactured water systems. However, several studies have reported that this procedure was ineffective at remediating water distribution systems as *Legionella* was able to maintain long term persistent contamination. Up to 25% of *L. pneumophila* cells survived heat treatment of 70°C, but all of these were in a viable but non-culturable state. This demonstrates the limitations of the culture method of *Legionella* detection currently used to evaluate disinfection protocols. In addition, it has been demonstrated that pasteurization and nutrient starvation can select for thermal tolerant strains, where *L. pneumophila* was consistently identified as having greater thermal tolerance compared to other *Legionella* species. This review demonstrates that further research is needed to investigate the effectiveness of pasteurization as a disinfection method. In particular, it focuses on the potential for pasteurization to select for thermal tolerant *L. pneumophila* strains which, as the primary causative agent of Legionnaires disease, have greater public health significance compared to other *Legionella* species.

## Introduction

Opportunistic pathogens linked to manufactured water systems have been identified as an increasingly significant public health issue (Falkinham et al., 2015a,b). In the United States, it has been estimated that the annual cost of healthcare related waterborne diseases is $430 million (Collier et al., 2012). One of the primary waterborne pathogens of concern are *Legionella* spp., which are the causative agents of Legionnaires disease, an atypical pneumonia, and Pontiac fever, an acute febrile illness (Bartram et al., 2007; Cunha, 2010; Falkinham et al., 2015a).

Transmission of *Legionella* to humans occurs through the inhalation of contaminated aerosols or aspiration of contaminated water (Blatt et al., 1993; Bartram et al., 2007; Cassier et al., 2013; Hines et al., 2014). Worldwide, incidences of Legionnaires’ disease are often linked to manufactured water systems (Beer et al., 2015) and in the United States, *Legionella* is the primary cause of all drinking water related outbreaks (Centers for Disease Control and Prevention, 2013). Common factors that enable *Legionella* persistence in water systems include, biofilm formation, growth in amoebae, growth in low nutrient environments and disinfectant resistance or tolerance (Ashbolt, 2015; Falkinham et al., 2015b).

Currently, there are over 60 described species of *Legionella;* however, worldwide the primary cause of Legionnaires disease is *L. pneumophila* (Bartram et al., 2007; Centers for Disease Control Prevention, 2011; Zhang et al., 2014). There are 15 *L. pneumophila* serogroups, among which serogroup 1 is the predominate cause of human infection (Victor et al., 2002). In the United States it has been estimated that nosocomially acquired *L. pneumophila* has a fatality rate between 25 and 48% (Mercante and Winchell, 2015; Soda, 2017). As a consequence of aging populations there will be an increase in vulnerable populations residing in healthcare facilities and hence there is a need to reevaluate current methods to control *Legionella* in engineered water systems (Mercante and Winchell, 2015; Whiley, 2016). One of the challenges when evaluating the effectiveness of *Legionella* control measures is that the standard detection method is culture, which cannot detect viable but non-culturable (VBNC) *Legionella* (Kirschner, 2016). This is especially concerning in manufactured potable water systems as low nutrients, high temperatures and disinfection residuals have all been shown to induce VBNC *Legionella* (Chang et al., 2007; Turetgen, 2008; Whiley and Taylor, 2016). This review will examine the literature relating to *Legionella* persistence in water distribution systems despite the implementation of pasteurization as a disinfection method. The potential for pasteurization to select for thermal tolerant strains will also be discussed.

## Thermal Disinfection of Manufactured Water Systems

It has been demonstrated that buildings with hot water distribution systems set below 60°C are more likely to be contaminated with *L. pneumophila* (Falkinham et al., 2015a). As such, many recommendations state that the temperature of healthcare hot water systems should be maintained above 55°C as part of routine control measures against *Legionella* (Health and Safety Executive [HSE], 2013; EnHealth, 2015; United States Environmental Protection Agency, 2015). However, this is a recommendation for the control but not the eradication of *Legionella* (United States Environmental Protection Agency, 2016).

Pasteurization (otherwise known as superheat and flush) has been identified as a potential disinfection method for remediating engineering water systems (Ashbolt, 2015). It is recommended that the process should involve raising the hot water temperature to 71–77°C so that the temperature reaches at least 65°C at the outlets. Outlets should then be flushed at this temperature for between 10 and 30 min (United States Environmental Protection Agency, 2016). Pasteurization of water distribution systems is often preferentially selected as a disinfection process due to the fact that no special equipment is needed and it can be implemented expeditiously (Chen et al., 2005). Previous studies have demonstrated the success of this process in reducing the number of *Legionella* present (Best et al., 1983; Zacheus and Martikainen, 1996; Darelid et al., 2002; Peiró Callizo et al., 2005). However, despite the successful decrease in *Legionella*, either complete elimination was not achieved (Darelid et al., 2002) or the system was quickly recolonized after thermal disinfection (Zacheus and Martikainen, 1996). Thermal treatment is an important *Legionella* control measure; however, it is essential that we fully understand its limitations in order to make informed risk management decisions.

## *In Situ* Studies Demonstrating *Legionella* Survival of Thermal Disinfection

There are several *in situ* studies that have demonstrated the ability of *Legionella* to survive thermal disinfection (Vervaeren et al., 2006; Chang et al., 2007; Farhat et al., 2010). In one, a pilot-scale hot water system was artificially contaminated with environmental microflora, including *Legionella* spp., to determine the efficacy of thermal disinfection (Farhat et al., 2010). Following two consecutive pasteurization treatments (70°C for 30 min) it was discovered that the initial treatment temporarily reduced the concentration of *Legionella*, but the second treatment did not affect either the *Legionella* concentration or the total number of bacteria present in the biofilm. An earlier study using a stagnant water model demonstrated that the growth of *L. pneumophila* present in a natural biofilm was stimulated when exposed to heat treated potable water (30 min at 60°C) (Vervaeren et al., 2006); this may explain the rapid recolonization of hot water systems after thermal treatments. The role of biofilm in *Legionella* survival was also illustrated by a recent study conducted in Israel. High concentrations of *Legionella* in water samples significantly correlated with the presence of *Legionella* in biofilm collected from potable water distribution systems in Israel (Rodríguez-Martínez et al., 2015).

Nutrient starvation can also influence the tolerance of *L. pneumophila* to pasteurization (Chang et al., 2007). *L. pneumophila* was nutrient starved (suspended in sterile ultrapure water and incubated at 37°C without CO_2_) for 1 or 14 days prior to thermal disinfection. Heat treatment of 30 min at 70°C caused all *L. pneumophila* to become VBNC (determined using stains to assess cells with intact membranes, consistent with viability). This demonstrated that 14% of 14 day and 5% of 1 day nutrient starved *L. pneumophila* survived the heat treatment. It was shown that the duration of *L. pneumophila* starvation was a statistically significant (*P* < 0.005) factor that adversely affected the percentage of *L. pneumophila* surviving heat treatment (Chang et al., 2007). Further research is required to determine if other factors that promote VBNC *Legionella*, such as chlorine or monochloramine disinfection, may influence the tolerance of *Legionella* to pasteurization (Dusserre et al., 2008). Another study by Allegra et al. (2008) similarly used viability stains (Syto9 to assess intact membranes and propidium iodide for damaged membranes) and flow cytometry to monitor *Legionella* which had become VBNC after pasteurization. It was found that for 6 of the 12 *Legionella* strains tested, 10–25% of the cells remained viable after heat treatment of 70°C for 30 min. *L. pneumophila* serogroup 1 was the most resistant with more than 15% of cells remaining viable after 1 h at 70°C. Similarly, Epalle et al. (2015) demonstrated that heat treatment (70°C for 30 min) resulted in *L. pneumophila* serotype 1 strains (clinical strain [Lp1-004], GFP-reference strain [Lp1-008] and an environmental strain [Lp1-RNN]) to be non-culturable, but 10–40% of cells still viable. The *L. pneumophila* Lp1-004 isolate had the highest percentage of cells with intact membranes and the viability of these VBNC cells was confirmed by demonstrating that they could still infect *Acanthamoeba polyphaga* (see below).

## Thermal Disinfection and *L. pneumophila* Persistence

Several studies monitoring manufactured water systems, have demonstrated the ability of *L. pneumophila* to maintain persistent colonization of manufactured water systems despite routine thermal disinfection (Perola et al., 2005; Scaturro et al., 2007; Allegra et al., 2011; Bédard et al., 2016). A recent investigation in Canada examined a hospital hot water system following a nosocomial *L. pneumophila* outbreak. Two separate water distribution systems within the hospital were disinfected using pasteurization. System A was heat treated once (70°C for 30 min) whereas system B was heat treated twice, 1 week apart. Using the culture method of detection it was demonstrated that *L. pneumophila* numbers were significantly reduced in system A but no reduction was observed in system B. Also despite maintaining weekly flushing, reducing pipe lengths and maintaining the water temperature above 55°C, low levels of *L. pneumophila* could not be eliminated (Bédard et al., 2016). Similarly, in Finland after an outbreak of *L. pneumophila* serogroup 5, it was found using culture that the hospital hot water distribution system was colonized with *L. pneumophila* serogroups 5 and 6. The system was disinfected by pasteurization of up to 80°C followed by flushing. However, long term eradication of the serogroup 5 strains was never achieved and only 1 of the serogroup 6 strains was not present after thermal treatment (Perola et al., 2005). In Italy after a nosocomial outbreak of *L. pneumophila*, molecular typing of the clinical isolate linked the outbreak to the hospital’s hot water system. The follow up investigation suggested that the *L. pneumophila* strain had maintained persistent contamination of the hot water system for 15 years (Scaturro et al., 2007). These studies demonstrate the inability of pasteurization to eradicate *L. pneumophila* in established manufactured potable water system.

## Role of Amoeba

*Legionella* is an opportunistic intracellular parasite of free living protozoa such as *Acanthamoebae* and *Vermamoeba* (Barbaree et al., 1986; Fields et al., 1989). This provides a mechanism to survive unfavorable environmental conditions, including heat treatment (Lau and Ashbolt, 2009). Thus, this opportunistic behavior may contribute to the long term persistence of *Legionella* in water distributions systems treated with thermal disinfection protocols (Ashbolt, 2015). Storey et al. (2004) demonstrated that *L. erythra*, and *L. pneumophila* replicating within free living *Acanthamoebae* had increased resistance to thermal treatment compared to planktonic *Legionella*. It was also demonstrated that the *Acanthamoebae* cyst remained viable after heat treatment of 80°C for 10 min suggesting that this method of thermal disinfection would be insufficient for the control of *Acanthamoebae* carrying *Legionella* in water distribution systems. Another study by Dobrowsky et al. (2016) demonstrated using viability PCR (vPCR) that both *Acanthamoeba* and *Legionella* were present in rainwater after pasteurization at high temperatures (68–93°C).

Other work has shown significant correlation between the presence of *Vermamoeba* spp. and *Legionella*. The positive link between thermal tolerance of *Legionella* and these amoebae species has also been noted (Rhoads et al., 2015; Lu et al., 2017; van der Kooij et al., 2017). Although *Vermamoeba* have been demonstrated to be less thermally tolerant than *Acanthamoebae*, their cysts exhibit much higher thermal tolerance then trophozoites. This may permit amoebic cysts to harbor *Legionella* species throughout a thermal disinfection event (Cervero-Aragó et al., 2014; Dobrowsky et al., 2016).

## Pasteurization Selects for Thermal Tolerant *Legionella pneumophila* Strains

One potential mechanism enabling *Legionella* to maintain long term contamination of water distribution systems is thermal tolerance (Storey et al., 2004). A longitudinal study in France isolated *L. pneumophila* and *L. anisa* strains from four hot water circuits of different hospital buildings over 20 years. Three of the hospital hot water circuits had undergone varying heat treatments of either 65°C for 24 h or 70°C for 30 min. The isolated strains from different circuits were not related; however, those isolated within the same circuit over time had identical genotypic profiles. After subjecting these strains to *in situ* heat treatment experiments of 30 min at 70°C, the mean percentage of survival ranged from 4.6 to 71.7%. The strains with the highest percentage of survival were isolated from hospital hot water circuits that were more frequently subjected to thermal disinfection procedures (Allegra et al., 2011).

Persistent contamination of a hospital hot water system by *L. pneumophila* serogroup 1 and serogroup 2 was also examined by Steinert et al. (1998). The hospital water system was pasteurized (70°C for 12 h with an initial 3 min flushing of all outlets); however, *Legionella* concentrations at selected locations returned to pre-pasteurization levels within 3 months. A follow-up heat disinfection saw regrowth to pre-pasteurization concentrations in only 2 months. Pulse field gel electrophoresis analysis of isolates revealed that they were identical for all isolates of the same serogroup suggesting survival of the heat disinfection process. Follow up temperature tolerance experiments demonstrated that the serogroup 1 strain had greater heat tolerance that the serogroup 2 strain. This is supported by Borella et al. (2005) who surveyed Italian hotel hot water systems and detected *L. pneumophila* in 45.8% of water samples. A risk analysis demonstrated that higher chlorine levels and higher temperatures were associated with higher risk for *L. pneumophila* serogroup 1 whereas the opposite was observed for serogroups 2–14. Similarly, a study of water distribution systems in Greece examined the presence of *Legionella* after two treatments of thermal disinfection (70–80°C for up to 3 days). It was demonstrated that after the first treatment 45% and after the second 9% of the water distribution systems still contained *Legionella*≥1000 colony forming units/L. It was also demonstrated that *L. pneumophila* was more heat resistant than other *Legionella* spp. (Mouchtouri et al., 2007). A study from Germany, examined the diversity of *Legionella* species in hot and cold water samples collected from the drinking water distribution system in the city of Braunschweig. It was demonstrated that the composition of *Legionella* species in cold water differed from that present in hot water. In the hot water samples, *L. pneumophila* was present during all seasons at relatively high abundances. It was also demonstrated that *Legionella* species (including *L. pneumophila*) which were detected in the hot water samples were able to grow at temperatures above 50°C and survive (or potentially grow) at temperatures up to 63°C (Lesnik et al., 2016).

It has been postulated that the thermal tolerance of *L. pneumophila* could be attributed to its heat shock response (Lema et al., 1988). Heat shock proteins are responsible for increasing bacterial tolerance to unfavorable environmental conditions and stressors by degrading and reactivating damaged proteins (Parsell and Lindquist, 1993). Li et al. (2015) demonstrated that *L. pneumophila* grown in water significantly up-regulated genes responsible for the production of heat shock proteins compared to *L. pneumophila* grown in a nutrient rich environment. This is supported by previous research that demonstrated that *Escherichia coli* heat shock proteins are induced by starvation (Jenkins et al., 1991). The up-regulation of genes encoding heat shock proteins could explain the results by Chang et al. (2007) that demonstrated nutrient starvation was a significant factor promoting thermal tolerance.

This has additional public health significance, as it has been suggested that heat shock proteins are involved in promoting *L. pneumophila* pathogenicity (Fernandez et al., 1996; Zhan et al., 2015). Surface-exposed heat shock protein Hsp60 has been demonstrated to promote attachment and invasion in a HeLa model cell line. It was shown that the invasiveness of *L. pneumophila* with defective *dot/icm* Type IV secretion system genes, (resulting in Hsp60 not being surface exposed) was reduced by approximately 1000-fold (Hoffman and Garduno, 1999). In addition, Hsp60 contributes to promoting mitochondria recruitment to the *Legionella* containing vacuole, which is where intracellular replication occurs (Chong et al., 2009). This suggests that Hsp60 is involved in both attachment and entry into a host cell as well as early development stages of the *Legionella* containing vacuole (Fernandez et al., 1996; Zhan et al., 2015).

This is supported by a recent study that demonstrated clinical *L. pneumophila* strains exhibited superior capacity for growth at higher temperatures (42°C) compared to environmental isolates. In contrast, at lower temperatures (25°C) the opposite was observed (Sharaby et al., 2017). The identified emergence of new disease-associate clones of *L. pneumophila* may, in part, be accelerated by selective pressures exerted by heat treatments for disinfection. More generally, this genetic diversification seems to be an adaptive response to the conditions unique to the built environment (David et al., 2016; Lesnik et al., 2016).

## Conclusion

More research is needed to explore the potential for pasteurization to select for thermal tolerant *Legionella* strains. Worldwide, *L. pneumophila* is the primary causative agent of Legionnaires disease and as such, pasteurization potentially selecting for *L. pneumophila* with increased thermal tolerance would have significant public health implications. Pasteurization may be appropriate for short term control of an identified colonization problem. However, its utility as an ongoing strategy for *Legionella* control is questionable. Future research is required to investigate whether pasteurization potentially selects for virulent strains or promotes increased virulence as part of the heat shock response. These studies will need to take into consideration the limitations of the culture method of detection given that previous research has demonstrated pasteurization to induce VBNC state. Given that VBNC *Legionella* may result in false negative culture results, this also has implications for the risk management of building water distribution systems that utilize culture detection to evaluate potential control strategies and disinfection protocols.

## Author Contributions

HW wrote first draft. MB and RB provided academic input and critical revision of the article. All authors approve the final version.

## Conflict of Interest Statement

The authors declare that the research was conducted in the absence of any commercial or financial relationships that could be construed as a potential conflict of interest.
